# Tick Cell Lines in Research on Tick Control

**DOI:** 10.3389/fphys.2020.00152

**Published:** 2020-02-25

**Authors:** Ahmed Al-Rofaai, Lesley Bell-Sakyi

**Affiliations:** Department of Infection Biology, Institute of Infection and Global Health, University of Liverpool, Liverpool, United Kingdom

**Keywords:** *Rhipicephalus microplus*, *Ixodes* spp., control, tick cell line, acaricide, resistance, metabolism, anti-tick vaccine

## Abstract

Ticks and the diseases they transmit are of huge veterinary, medical and economic importance worldwide. Control of ticks attacking livestock and companion animals is achieved primarily by application of chemical or plant-based acaricides. However, ticks can rapidly develop resistance to any new product brought onto the market, necessitating an ongoing search for novel active compounds and alternative approaches to tick control. Many aspects of tick and tick-borne pathogen research have been facilitated by the application of continuous cell lines derived from some of the most economically important tick species. These include cell lines derived from acaricide-susceptible and resistant ticks, cell sub-lines with *in vitro*-generated acaricide resistance, and genetically modified tick cells. Although not a replacement for the whole organism, tick cell lines enable studies at the cellular and molecular level and provide a more accessible, more ethical and less expensive *in vitro* alternative to *in vivo* tick feeding experiments. Here we review the role played by tick cell lines in studies on acaricide resistance, mode-of-action of acaricides, identification of potential novel control targets through better understanding of tick metabolism, and anti-tick vaccine development, that may lead to new approaches to control ticks and tick-borne diseases.

## Introduction

Ticks are obligate, haematophagous ectoparasites. Like mosquitoes, they cause problems to their hosts both from skin irritation and damage during blood feeding and by transmitting numerous pathogens including viruses, bacteria and protozoa. Consequently, ticks and tick-borne pathogens (TBPs) are of huge veterinary, medical and economic importance due to their negative impact on the health of humans and domestic animals (de la Fuente et al., 2008). Therefore, many control strategies have been devised to eliminate or avoid ticks and reduce the spread of TBPs, such as personal protection, landscape management, chemicals, vaccination, and biological control (Stafford et al., 2017). Amongst these, chemical control using a variety of acaricidal compounds is still the main strategy for controlling ticks affecting livestock and companion animals. However, many tick species develop resistance to these chemicals, which has led to attempts to find natural alternatives by studying the effects of some natural products on ticks and TBPs (Rodriguez-Vivas et al., 2018). Globally, the most economically important species, the invasive Asian cattle tick *Rhipicephalus* (*Boophilus*) *microplus*, is also the most notorious for rapid development of resistance to every new acaricidal product brought onto the market (Jonsson and Hope, 2007; Abbas et al., 2014). Acaricide-resistant *R. microplus* are widespread throughout tropical and sub-tropical Asia, Latin America and Australia (Abbas et al., 2014), and are rapidly spreading in Southern, Central and West Africa (Nyangiwe et al., 2018; Silatsa et al., 2019).

Tick cell lines have an invaluable role to play in many aspects of tick and TBP research including tick biology, host-vector-pathogen relationships, genetic manipulation, genomics and proteomics (Bell-Sakyi et al., 2007). In addition, tick cell lines play a major role as essential tools in *in vitro* studies to examine and assess the impact of chemical molecules and vaccines against ticks and TBPs, as well as to investigate the mechanisms of resistance and tick metabolism which can lead to the development of novel approaches to control ticks and TBPs (Bell-Sakyi et al., 2018). While >60 continuous cell lines have now been established from 16 ixodid and three argasid tick species (Bell-Sakyi et al., 2018), studies on tick and pathogen control have predominantly centered around cell lines derived from, not surprisingly, *R. microplus*. A smaller number of studies have used cell lines derived from other tropical *Rhipicephalus* species of veterinary importance, from *Ixodes ricinus*, a European tick that feeds on a wide range of domestic and wild animal hosts as well as attacking humans, and from the medically important North American species *Ixodes scapularis* (Table 1).

**TABLE 1:** Tick cell lines used in studies on tick control reviewed in this article.

Tick species	Distribution	Economic importance	Cell line	Geographic origin of parent ticks	Original references
*Rhipicephalus microplus*	Worldwide tropical and sub-tropical	Serious ectoparasitic pest of cattle; transmits anaplasmosis and babesiosis; high propensity to develop acaricide resistance	BmVIII-SCC	Mexico	Holman, 1981
			BME26	Mexico	Kurtti et al., 1988
			BME/CTVM2	Costa Rica	Bell-Sakyi, 2004
			BME/CTVM5	Colombia	Bell-Sakyi et al., 2007
			BME/CTVM6	Colombia	Bell-Sakyi, 2004
			BME/CTVM23	Mozambique	Alberdi et al., 2012
			BME/CTVM30	Mozambique	Alberdi et al., 2012
*Rhipicephalus appendiculatus*	East, Central and Southern Africa	Transmits East Coast fever of cattle and Nairobi sheep disease	RAN/CTVM3	Kenya	Bekker et al., 2002
			RA243	East Africa	Varma et al., 1975
*Rhipicephalus evertsi*	Sub-Saharan Africa	Transmits equine babesiosis, bovine anaplasmosis and causes tick toxicosis in ruminants	REN/CTVM32	South Africa	Bell-Sakyi et al., 2015
*Rhipicephalus sanguineus*	Worldwide tropical, sub-tropical and temperate	Transmits canine babesiosis, ehrlichioses and human rickettsioses	RSE/PILS35	France	Koh-Tan et al., 2016
			RML-RSE	United States	Yunker et al., 1981; Bell-Sakyi et al., 2015
*Ixodes ricinus*	Europe	Transmits borreliosis, neoehrlichiosis, anaplasmosis, babesiosis, tick-borne encephalitis, louping ill	IRE/CTVM19	United Kingdom	Bell-Sakyi et al., 2007
			IRE/CTVM20	United Kingdom	Bell-Sakyi et al., 2007
*Ixodes scapularis*	North America	Transmits Lyme borreliosis, human anaplasmosis and babesiosis, Powassan encephalitis	IDE8	United States	Munderloh et al., 1994
			ISE6	United States	Kurtti et al., 1996

This Mini Review focuses on the role of tick cell lines in studies on control of ticks and the few studies dealing with approaches to combined control of ticks and TBPs. While tick cell lines have been used in many studies contributing to aspects of pathogen control *per se*, these are beyond the scope of our review. Here we review selected studies to highlight how tick cell lines can contribute to tick control research, bearing in mind that results gained *in vitro* should be validated *in vivo*.

## Tick Cell Lines in Studies on Acaricide Resistance

Tick cell lines have been investigated for their potential as cheap and ethical tools to study acaricide resistance. Some studies focused on establishing drug-resistant cell lines which provide a useful tool to study and understand the mechanisms of resistance, an important step toward improving detection and prevention of tick resistance against acaricides. Tick cell lines are also a useful resource for identifying genes responsible for acaricide resistance and some published studies have focused on this aspect.

### Generation and Application of Acaricide-Resistant Tick Cell Lines

Cossio-Bayugar et al. (2002b) generated three organophosphate-resistant *R. microplus* cell sub-lines by exposing the *R. microplus* BmVIII-SCC cell line (Holman, 1981), originally derived from embryos of acaricide-susceptible ticks, to gradually increasing concentrations of the organophosphate compound coumaphos (Table 2). They then assessed the levels of esterase, an enzyme that, when present at high levels, is responsible for causing resistance by modifying the organophosphate target site to be less sensitive. After exposure to coumaphos, there were higher levels of esterase in the resistant sub-lines compared to the original susceptible cell line. These findings, which were in line with previous *in vivo* studies (Miranda et al., 1995; Rosario-Cruz et al., 1997), validated the use of tick cell lines to study development of resistance in ticks, which could thereafter help to determine a suitable approach to formulating acaricides that would be less likely to induce resistance.

**TABLE 2:** Acaricides and pesticides used with tick cell lines in studies on tick control reviewed in this article.

Name	Chemical class	Route of application to animals	Mode of action
Amitraz	Amidines	Spot-on, spraying and dipping	Has an antagonistic effect on octopaminergic G protein-coupled receptors of the nerve cells causing paralysis and hyperexcitation in ticks
Coumaphos	Organophosphate compounds	Spraying and dipping	Acts on the nervous system as an inhibitor of transmission of nervous signals causing paralysis in the parasite
Fipronil	Phenylpyrazole compounds	Pour-on, spot-on and spraying	Inhibitor of GABA (gamma-aminobutyric acid), a key neurotransmitter in the central nervous system.
Ivermectin	Avermectins (macrocyclic lactones)	Pour-on, orally and by injection	Acts as inhibitor of GABA causing neuromuscular blockage, also opens chloride ion channels in membranes of the nervous system and further depresses neuronal function.
Paraquat	Dipyridylium (viologen)	Not applied to animals	A highly toxic herbicide known to induce oxidative stress in cells because of its ability to induce generation of superoxide ions
Permethrin	Synthetic pyrethroids	Pour-on, spot-on, spraying and dipping	Acts on the membrane of nerve cells blocking the closure of the ion gates of the sodium channel during re-polarization, and thereby disrupts transmission of nervous impulses.

This group then used the coumaphos-resistant *R. microplus* cell sub-lines to evaluate the mechanisms that may contribute to acaricide resistance by further characterizing them compared to susceptible control cells (Cossio-Bayugar et al., 2002a). In addition to higher esterase levels, they found that the resistant cells exhibited higher levels of intracellular calcium and glutathione, decreased glutathione S-transferase activity, and reduced plasma and mitochondrial membrane potentials. Their results were consistent with the *in vivo* observations of increased esterase activity in organophosphate-resistant ticks, as well as resistance mechanisms found in other cell systems. One of the coumaphos-resistant *R. microplus* cell sub-lines played an important role in a further study (Cossio-Bayugar et al., 2005) that identified amino acid substitutions in the protein encoded by the phospholipid hydroperoxide glutathione peroxidase (PHGPx) gene in susceptible and resistant *R. microplus*, highlighting the possibility of its relationship with acaricide resistance in these tick populations.

A study conducted by Pohl et al. (2014) also focused on understanding the mechanisms of acaricide resistance in *R. microplus*. In this study, the authors established an ivermectin-resistant sub-line (BME26-IVM) of the *R. microplus* embryo-derived cell line BME26 (Kurtti et al., 1988; Esteves et al., 2008) by exposing susceptible cells to increasing concentrations of ivermectin (Table 2) over 46 weeks. Using the BME26-IVM sub-line, the contribution of the ATP-binding cassette (ABC) transporter mechanism was investigated by evaluating mRNA expression using quantitative RT-PCR. This study demonstrated increased levels of ABC transporter gene transcripts in the ivermectin-resistant cells compared to the original susceptible BME26 cells.

### Effect of Acaricide Treatment of “Susceptible” Tick Cell Lines on Expression of Genes Associated With Resistance

Taking a different approach and using a cell line expected to be susceptible to ivermectin, Mangia et al. (2016) evaluated the expression of selected members of the ABC transporter subfamily B genes encoding P-glycoproteins (PgPs) in the *I. ricinus* embryo-derived cell line IRE/CTVM19 following treatment with ivermectin. The authors showed that ABC pump expression was not significantly modulated by ivermectin treatment, and expression of one of the subfamily genes was not detected. Interestingly, this study revealed that the IRE/CTVM19 cell line was able to tolerate a much higher concentration of ivermectin (30 μg/ml) than the *R. microplus*-derived line BME26 that did not survive after exposure to 12.5 μg/ml ivermectin (Pohl et al., 2014). This could have been due to biological differences between the two tick species from which the cell lines were derived, differences in the phenotypic composition of the cell lines and/or in the culture conditions used. This should be considered when conducting such *in vitro* experiments using tick cell lines.

In a further study, Mangia et al. (2018) treated IRE/CTVM19 cultures with different concentrations of the acaricides permethrin, amitraz and fipronil (Table 2). They assessed cell viability, morphology, metabolic activity and expression of ABC genes at day 10 post-treatment. In general, the results showed that fipronil had the greatest significant negative effects on cell viability and metabolic activity, followed by permethrin, while amitraz only caused a significant decrease in these parameters at the highest concentration tested. The negative effects of fipronil and permethrin were confirmed by their effects on ABC gene expression with upregulation of ABCB6, ABCB8, and ABCB10, while amitraz had little or no effect on expression of any of the ABC genes tested.

### Exploiting Tick Cell Lines Derived From Susceptible and Resistant Ticks to Identify Genes Involved in Acaricide Resistance

For some of the currently available *R. microplus* cell lines, the acaricide resistance status of the parent ticks from which the cultures were derived is known. As already mentioned, the parent BmVIII-SCC and BME26 cells used by, respectively, Cossio-Bayugar et al. (2002b) and Pohl et al. (2014) to derive acaricide-resistant sub-lines *in vitro* were originally susceptible to the compounds used, implying that the parent ticks of both lines, originally from Mexico, were also acaricide-susceptible. Two additional *R. microplus* cell lines, BME/CTVM2 (Bell-Sakyi, 2004) and BME/CTVM4 (Bell-Sakyi et al., 2007), were derived from known acaricide-susceptible ticks from Costa Rica, while two other cell lines, BME/CTVM5 (Bell-Sakyi et al., 2007) and BME/CTVM6 (Bell-Sakyi, 2004) were derived from Colombian ticks known to be resistant to organophosphates, organochlorines and amitraz (Koh-Tan et al., 2016). While these cells whose natural resistance status derives from their parent ticks offer a potentially very interesting complement to the *in vitro*-derived resistant *R. microplus* cell lines of Cossio-Bayugar et al. (2002b) and Pohl et al. (2014), to date they have only been exploited in a single study. Koh-Tan et al. (2016) investigated the expression of the β-adrenergic octopamine receptor (βAOR) gene, associated with amitraz resistance, and the ATP-binding cassette B10 (ABCB10) gene, associated with macrocyclic lactone resistance, in tick cell lines derived from *R. microplus*, including BmVIII-SCC, BME/CTVM2, BME/CTVM5 and BME/CTVM6, *Rhipicephalus appendiculatus*, *Rhipicephalus sanguineus*, and *Rhipicephalus evertsi* (Table 1). Expression of the βAOR gene was detected in all the *Rhipicephalus* spp. cell lines, but in BME/CTVM6, derived from amitraz-resistant ticks, a novel larger amplicon was detected. This was found to encode a 12-amino acid insertion identified as a duplication of the immediately upstream sequence; the insertion was also present in genomic DNA from BME/CTVM5 cells, similarly derived from amitraz-resistant ticks, but transcription of the novel amplicon was not detectable. Expression of the ABC10 gene was approximately seven-fold higher in BME/CTVM6 cells compared to all other cell lines examined including BME/CTVM5. These findings suggest that BME/CTVM6 cells in particular could be a useful model system for studying resistance to multiple acaricide groups in *R. microplus*; further experiments are needed to determine whether additional genes are mutated in this cell line.

## Tick Cell Lines Elucidate Novel Aspects of Tick Cell Metabolism to Identify Potential Control Targets

Tick cell lines have also played a valuable role in the study of tick cell metabolism which can help in developing novel control methods. De Abreu et al. (2013) investigated the role of protein kinase B (AKT) and glycogen synthase kinase 3 (GSK3) in glycogen metabolism and cell viability using the *R. microplus* cell line BME26, and the results revealed an antagonistic role for AKT and GSK3. A reduction in AKT caused a decrease in glycogen content, cell viability and altered cell membrane permeability, whereas the inhibition of GSK3 promoted an increase in glycogen content.

To better understand energy metabolism during embryonic development in ticks, Da Silva et al. (2015) used the BME26 cell line to evaluate several genes involved in gluconeogenesis, glycolysis and glycogen metabolism, the major pathways for carbohydrate catabolism and anabolism. Their results showed that several genes displayed mutual regulation in response to glucose treatment, and the transcription of gluconeogenic genes in tick cells is controlled by GSK3.

The BME26 cell line also played an essential and effective role in the first report on characterization of a cell cycle protein in arachnids, and the reversal of its functions with an inhibitor (Gomes et al., 2013). The BME26 cell line was used in an *in vitro* inhibition assay to determine the inhibitory effects of roscovitine, a purine derivative, on tick cyclin-dependent kinases (CDKs), which are essential for cell cycle progression, and the results suggested that CDKs may present an alternative strategy for designing a novel acaricide that targets both oogenesis and embryogenesis processes.

The *I. scapularis* embryo-derived tick cell line ISE6 (Kurtti et al., 1996) is the one of the most widely used tick cell lines due to its susceptibility to a wide range of TBPs (Oliver et al., 2015). ISE6 cells were used by Cabezas-Cruz et al. (2017) to study possible control of infection with the obligate intracellular bacterium *Anaplasma phagocytophilum*, causative agent of disease in humans, ruminants, horses and dogs, by increasing the synthesis of phosphoenolpyruvate from tyrosine. Ferritins (FER) are iron-storage proteins that act to store excess iron available in the cellular iron pool by the binding and oxidation of the ferrous ion (Fe2+) and the formation of ferric ion (Fe3+) in the FER core cavity (Galay et al., 2015). Hernandez et al. (2018) were able to induce intracellular ferritin (FER1) protein expression by exposing ISE6 cells to various concentrations of ferrous sulfate. They showed that silencing of FER1 expression by RNA interference led to a decrease in ISE6 cell proliferation and an increase in mortality, concluding that ISE6 cells could be a good tool to further understanding of the mechanism of FER1 action. Thereafter, Hernandez et al. (2019) characterized the activity of an iron-inducible tick promoter derived from the tick *Haemaphysalis longicornis*, previously identified by Kusakisako et al. (2018b), in ISE6 cells, which could be a valuable tool in the development of a gene-manipulation system to control ticks and TBPs. Kusakisako et al. (2018a) established methods to visualize H_2_O_2_ in ISE6 cells using an intracellular H_2_O_2_ probe and observed that paraquat, known to induce oxidative stress in mammalian cells (Table 2), was also effective in this respect in ISE6 tick cells. Iron derived from host blood may react with oxygen in the tick’s body and lead to high levels of H_2_O_2_, causing oxidative stress such as DNA strand breaks, lipid peroxidation and degradation of other molecules (Kusakisako et al., 2018a; Hernandez et al., 2019).

## Genetic Manipulation of Tick Cell Lines

The development of new strategies for control of ticks could be greatly enhanced by exploring genetic manipulation of ticks and tick cell lines. Demonstrating proof of concept, Kurtti et al. (2006) first reported stable transfection of the *I. scapularis* cell line ISE6 with a gene encoding a fluorescent protein using the Sleeping Beauty transposon system. In a different approach, Cossio-Bayugar and Miranda-Miranda (2007) reported retroviral transfection of primary *R. microplus* cell cultures with green fluorescent protein; fluorescence was detectable for up to 2 weeks following retroviral transfection. Using yet another approach, Machado-Ferreira et al. (2015) achieved the expression of transgenes in *R. microplus* ticks and the cell line BME/CTVM2 using *Agrobacterium tumefaciens* T-DNA constructs; evidence of transcription and protein synthesis were detectable for 2–3 weeks by fluorescence microscopy, western blot analyses and RT-PCR. These studies demonstrate that genetic manipulation approaches used with other animal cell systems can equally be applied to tick cell lines, and raise the possibility of modifying ticks to render them incapable of developing acaricide resistance.

## Tick Cell Lines Contribute to Anti-Tick Vaccine Development

Vaccination with recombinant tick antigens represents a promising tick control strategy. Vaccines containing recombinant tick antigens demonstrated advantages including reduction in acaricide application, cost-effectiveness and reducing the prevalence of tick-borne diseases through reducing exposure of cattle to infected ticks. However, the efficacies of these vaccines vary considerably between tick species and geographic location (de la Fuente et al., 2007). Tick cell lines have played a central role in identifying tick protective antigens to produce a variety of vaccines that contribute to the prevention and control of tick infestations and TBP infections. For example, three embryo-derived tick cell lines, *R. microplus* BME/CTVM2 and *I. scapularis* IDE8 (Munderloh et al., 1994) and ISE6, were used in a study conducted by Antunes et al. (2014) to identify tick proteins involved in tick-pathogen interactions. The aim was to identify candidate protective antigens that could be used as vaccines for the control of cattle infestations with *R. microplus* ticks and infection with the TBPs *Anaplasma marginale* and *Babesia bigemina* transmitted by *R. microplus*. As an important part of the experiment, antibodies produced against the *R. microplus* protein subolesin were tested using immunofluorescence and showed positive reactions in the three cell lines, but no effect was observed on pathogen DNA levels. In contrast, using the *I. scapularis* cell line ISE6 and the *I. ricinus* cell line IRE/CTVM20 (Bell-Sakyi et al., 2007), Contreras et al. (2017) determined that two candidate tick-protective antigens – *I. scapularis* lipocalin (ISCW005600) and lectin pathway inhibitor (AAY66632), and their *I. ricinus* homologs – constitute candidate protective antigens for the control of tick infestations as well as *A. phagocytophilum* infection. They showed that anti ISCW005600 and AAY66632 IgG significantly increased the percentage of apoptotic tick cells and decreased pathogen infection, which could help in the development of effective anti-tick and anti-pathogen vaccines.

## Conclusion

Ticks and TBPs have huge negative impacts on global livestock and public health and national economies. Scientific research has focused on creating and developing a variety of strategies to prevent and control ticks and TBPs. Tick cell lines represent essential tools in this area of research: *in vitro*-generated acaricide-resistant cell lines, cell lines derived from acaricide-resistant and susceptible ticks, genetically modified cell lines and cell lines supporting growth of intracellular TBPs all have a role to play. Experimental parameters can be easily controlled, effects at the cellular and molecular level can be determined, and cell lines reduce, while not ultimately replacing, the need for expensive *in vivo* studies involving maintenance of tick colonies and feeding on laboratory animals. As the numbers of cell lines derived from economically important tick species increase, so do the opportunities for exploiting them in a wide range of studies. For example, the potential for developing high-throughput preliminary screens of novel, potentially acaricidal compounds using cell lines from multiple tick species remains unexplored; such screens could greatly increase the number of active compounds identified for further testing at relatively low cost, and could be applied both to compound libraries held by pharmaceutical companies and to natural plant products. It is anticipated that gene editing of tick cell lines by technologies such as CRISPR will add another facet to the control portfolio. The Tick Cell Biobank (Bell-Sakyi et al., 2018), which houses all the cell lines mentioned in this review as well as cell lines derived from many other species of veterinary and medical concern, will continue to underpin research on tick and TBP control.

## Author Contributions

AA-R drafted the manuscript, LB-S edited the draft and both authors produced and agreed the final version.

## Conflict of Interest

The authors declare that the research was conducted in the absence of any commercial or financial relationships that could be construed as a potential conflict of interest. The reviewer UM declared a past co-authorship with one of the authors LB-S to the handling Editor.
